# Variations of care quality for infectious pulmonary tuberculosis in Taiwan: a population based cohort study

**DOI:** 10.1186/1471-2458-7-107

**Published:** 2007-06-11

**Authors:** Wei-Sheng Chung, Ray-E Chang, How-Ran Guo

**Affiliations:** 1 Hualien General Hospital, Department of Heath, Executive Yuan, Hualien, Taiwan; 2 Graduate Institute of Health Care Organization Administration, College of Public Health, National Taiwan University, Taipei, Taiwan; 3 Department of Environmental and Occupational Health, College of Medicine, National Cheng Kung University Hospital, National Cheng Kung University, Tainan, Taiwan; 4 Department of Occupational and Environmental Medicine, National Cheng Kung University Hospital, National Cheng Kung University, Tainan, Taiwan; 5 No. 17, Shu-Chow Rd., Room 639, Taipei 100, Taiwan

## Abstract

**Background:**

Effective and efficient care is required to prevent the spread of infectious pulmonary tuberculosis (PTB). We attempted to compare care quality among different healthcare institutions in Southern Taiwan.

**Methods:**

This study conducted population-based retrospective cohort design. One tuberculosis sanatorium, 2 medical centers, 11 regional hospitals, and 15 district hospitals and primary practitioners in the study area had reported tuberculosis cases, registered from January 1 to June 30 2003. Those cases with sputum positive PTB were followed 15 months after anti-tuberculosis treatment initiation. Meanwhile, Level of conformance with diagnostic guidelines, efficiency of diagnostic and treatment process, and treatment were measured as main outcome. Association was investigated using Chi-square tests, Kruskal Wallis tests, Mann-Whiteney U tests, and multiple logistic regression analysis to evaluate outcome differences among different levels of institutions.

**Results:**

The analyses included 421 patients. In comparison with patients receiving treatment at medical centers, regional hospitals, and district hospitals/primary practitioners, patients at the Chest Specialty Hospital were more likely to provide at least three sputum specimens (74.1% vs. 48.2%, 36.8%, and 50.0%), shorter workdays examining sputum smears (2.4 ± 2.4 days vs. 2.6 ± 2.1, 4.5 ± 3.1, and 3.5 ± 2.6 days), shorter interval between the first consultation and treatment (10.1 ± 18.3 days vs. 31.0 ± 53.6, 31.2 ± 70.4, and 25.4 ± 37.6 days), and a higher successful treatment rate (92.6% vs. 65.2%, 63.9%, and 68.0%). Furthermore, after adjusting age and gender, the patients treated by the pulmonologists and treated at Chest Specialty Hospital had significantly more successful treatment rate, of which odds ratios were 1.74 and 4.58 respectively.

**Conclusion:**

Differences in care quality exist among different types of healthcare institutions and among individual physicians. The implementation of practice guidelines should contribute to an improvement in the care quality of the treatment and diagnosis of PTB.

## Background

Pulmonary tuberculosis is a global problem currently resulting in the death of approximately 2 million people each year. If more stringent control is not implemented, approximately 1 billion people will become newly infected, 150 million people will become symptomatic, and 36 million will die from the disease between 2002 and 2020 [1].

For the past several decades, Taiwan committed itself to reduce the epidemic of tuberculosis. The mortality rate from tuberculosis has decreased from more than 100 cases per 100,000 people in the early 1950s to 5.8 cases per 100,000 people in 2003. However, in recent years, newly infected tuberculosis cases appear to be on the increase; the incidence rate has increased from approximately 50 cases per 100,000 in the early 1990s to more than 60 per 100,000 since the late 1990s [2]. Tuberculosis remains the leading infectious cause of death in Taiwan. The control of tuberculosis remains one of the major challenges faced by Taiwan's health care administration.

Taiwan's health care system has been significantly reformed in recent years. Two reform policies, which have profoundly influenced tuberculosis care are: (1) the introduction of the National Health Insurance and (2) the restructure of the tuberculosis control system. In 1995, Taiwan implemented a compulsory universal health insurance program for its residents. This program maintains a contract with more than 90% of Taiwan's health care institutions and without a referral system, insured persons are provided with complete freedom regarding choice of physician [3]. Thus, all patients with tuberculosis can freely seek medical aid at any healthcare institution from offices of primary practitioners to hospitals.

Previously, Taiwan adopted a dedicated tuberculosis control system with an extensive network reaching each township. This system effectively contained the spread of tuberculosis. However, during the late 1990s, the government-owned health care institutions underwent a dramatic reorganization. The tuberculosis control system was merged into the general health care system. Four sanatoria institutions located in northern, central, and southern Taiwan were reduced to one. In 2001, the Chest Specialty Hospital was the only remaining tuberculosis sanatorium. These two influencing policies have resulted in an increasing trend in the number of tuberculosis patients treated by institutions other than tuberculosis sanatoria. By 2001, only 10% of all patients with tuberculosis were reported by tuberculosis sanatoria [4]. However, at the same time, the number of newly reported patients had increased, with a reporting rate of 75 cases per 100,000 people in 2002. Moreover, the cure rate for newly diagnosed pulmonary tuberculosis patients was 75%, which is lower than the 85% target set by the World Health Organization (WHO) [5].

To effectively contain the spread of tuberculosis, the availability of quality medical care to patients is essential. The efficiency of the diagnosis process and effective treatment for cases with infectious pulmonary tuberculosis (PTB) are important factors. Therefore, the purpose of this study was to evaluate the quality of diagnosis and treatment at different levels of healthcare institutions in Taiwan.

## Methods

A population-based medical record review in southern Taiwan, where the study perimeters included four administrative areas: Chiayi County, Chiayi City, Tainan County, and Tainan City, covering 2.7 million residents, was performed. As authorized by law in Taiwan, all suspected and confirmed tuberculosis cases should be reported in a timely manner to the national computerized registry, maintained by the Center for Disease Control (CDC). Reporting of cases has been encouraged and reinforced through the implementation of a no-notification-no-reimbursement policy and the notification-for-fee policy since 1997 [6]. We requested data on all suspects and confirmed tuberculosis patients residing in the studied areas from the registry for the period January 1 to June 30, 2003.

Site visits to hospitals were arranged to review the medical records of each patient. Prior to these visits, the team, including four registered nurses (each with a minimum of 6 years clinical experience), two chief nurses (each with a minimum of 12 years clinical experience), and one pulmonologist, had received a series of training courses. The training was designed to ensure proper validation of data consistency. Medical records of the diagnostic and treatment processes were reviewed.

### Infectious PTB

Infectious PTB is referring to sputum culture-confirmed disease caused by Mycobacterium tuberculosis or two sputum smear examinations positive for acid-fast bacilli [7].

### Healthcare institutions

The institutions that had reported cases in the study area included the Chest Specialty Hospital (the only remaining tuberculosis sanatorium in Taiwan, which is geared towards specialized thoracic disease care, mainly for tuberculosis), 2 medical centers, 11 regional hospitals, and 15 district hospitals and primary practitioners (district hospitals and primary practitioners are regarded as at the same level in terms of the treatment of tuberculosis). In addition to inpatient services, hospitals in Taiwan provide considerable outpatient services, accounting for approximately 50% of their revenues, and are accredited by three levels. Medical centers, housing over 500 acute staffed beds, are designated to assume the responsibilities of providing healthcare services, training medical professionals, and conducting research. Regional hospitals have no less than 250 acute beds and are staffed with physicians of various specialties with the purpose of providing healthcare services to patients and training for specialists. District hospitals are designated to provide primary healthcare services, which are similar to those offered by primary practitioners in clinics providing outpatient services.

### Quality of care

Quality of care was measured using three groups of indicators: the level of compliance with diagnostic guidelines during diagnosis, the efficiency of diagnostic and treatment processes, and the outcome of treatment.

The diagnostic process should include a complete examination of the patient's symptoms, chest X-ray, sputum smear and cultures. Amongst the diagnostic quality indicators were the percentage of successful collection of at least three sputum specimens from the patients at the institution [8-10], and the percentage of patients with a positive sputum smear who demonstrated negative sputum culture results, which might result from having received anti-tuberculosis treatment or laboratory errors [11,12]. For the timeliness of the diagnostic and treatment process, the number of days, between the chest X-ray examination and the hospital visit, between the sputum examination and the hospital visit, laboratory diagnostic delay and healthcare institution delay were calculated. The laboratory diagnostic delay was defined as the number of workdays required for reporting sputum smear examination results, and the healthcare institution delay was defined as the number of days from the first related hospital visit to the initiation of anti-TB medication.

Indicators of the outcome of treatment included the successful treatment rate and the fatality rate. The patients who received successful treatment indicated that they had been cured or had received complete treatment. A cured case was defined as a patient with PTB who had completed treatment with a negative bacteriology result during the course of treatment and at the end of treatment. A case with a completed treatment was defined as a patient with PTB who had finished treatment, but with no bacteriology result at the end of treatment. A fatality case referred to a patient whose death occurred for any cause during the course of treatment [13].

### Ethical consideration

The CDC approved the present study. All staff involved in the study signed a letter of agreement to maintain patient confidentiality.

### Data analysis

Chi-square tests were performed to evaluate the differences in proportions of dichotomous and categorical variables. Since the distributions of indicators of timeliness were skewed, Kruskal Wallis tests were performed to evaluate the differences in these indicators among different levels of institutions. In addition Mann-Whitney U tests were used to compare two individual groups of sampled data. A multiple logistic regression was constructed using successful treatment or fatality of TB as the dependent variable. Independent variables include age, gender, treatment by the pulmonologist, and treatment at the Chest Specialty Hospital. Odds ratio (OR) and 95% confidence interval (CI) of it were used to estimate the effects of independent variables on successful treatment and fatality. All analyses were conducted using SPSS 10.0 statistical software, and all statistical tests were performed at the two-tailed significance level of 0.05.

## Results

From January 1 to June 30 2003, 491 patients with TB, who showed at least two positive sputum smears or a positive culture, were reported in the study area. Among these patients, the medical records of 482 (98%) patients were reviewed. After reviewing medical records, 61 patients were excluded from the study; including one foreign laborer who was deported after diagnosis, 11 patients with extrapulmonary tuberculosis, 38 misdiagnosed patients and 11 patients without any documented positive sputum smears or positive cultures. Therefore, 421 patients, 311 male (73.9%) and 110 female (26.1%), were included in the analyses. Patient age at diagnosis ranged from 18 to 95 years, (mean, 66 years), and the number of patients increased with age (13 below 25 years of age, 35 from 25 to 44 years of age, 110 from 45 to 64 years of age, and 263 above 64 years of age). The majority (95.0%) of patients were symptomatic, and the most common symptoms observed were cough (78.8%), fever (41.7%), and dyspnea (29.3%). The most common co-morbidity observed was diabetes (34.3%). No cases of AIDS were observed.

While 134 (31.9%) out of the 420 patients revealed cavitations on chest X-ray examinations, one patient (0.2%) showed normal findings. Nearly one-half (201; 47.7%) of patients had provided three or more sputum specimens, and 392 (93.1%) presented sputum cultures for mycobacteria. Fifteen months after treatment was initiated, the rate of successful treatment among patients who underwent complete follow-up was 68.9%.

Fifty-four patients (12.8%) were treated at the Chest Specialty Hospital, 112 (26.6%) at medical centers, 155 (36.8%) at regional hospitals, and 100 (23.8%) at district hospitals or by primary practitioners (Table 1). The proportion of patients treated by pulmonologists also varied across different levels of healthcare institutions (*p *< 0.001), with the Chest Specialty Hospital containing the highest proportion of patients.

**Table 1:** Comparison of care quality at different levels of institutions

	Medical center (n = 112)	Regional hospital (n = 155)	District hospital/primary practitioners (n = 100)	Chest specialty hospital (n = 54)	*P*-value
Diagnostic physician^a^; n (%)					< 0.001
Pulmonologist	76 (67.9)	118 (76.1)	67 (67.0)	53 (98.1)	
Non-pulmonologist	36(32.1)	37 (23.9)	33 (33.0)	1 (1.9)	
Quality of diagnosis; n (%)					
Sputum specimens for diagnosis^a^					< 0.001
<3 specimens	58(51.8)	98 (63.2)	50 (50.0)	14 (25.9)	
≥ 3 specimens	54 (48.2)	57 (36.8)	50 (50.0)	40(74.1)	
Positive sputum smear but negative sputum culture results^a^					< 0.001
Yes	1 (0.9)	12 (7.7)	19 (19.0)	2 (3.7)	
No	111 (99.1)	143 (92.3)	81 (81.0)	52 (96.3)	
Timeliness (mean ± SD)					
Days between chest X-ray examination and the hospital visit^b ^(n = 415)	1.8 ± 6.0	1.8 ± 5.6	1.7 ± 3.4	1.0 ± 0.0	0.044
Days between the sputum examination and the hospital visit^b ^(n = 415)	4.9 ± 13.3	4.1 ± 8.2	4.8 ± 14.7	1.5 ± 2.9	< 0.001
Healthcare institution delay^1,b ^(n = 378)	31.0 ± 53.6	31.2 ± 70.4	25.4 ± 3 7.6	10.1 ± 18.3	< 0.001
Positive AFB smear^b ^(n = 235)	11.2 ± 16.2	13.4 ± 31.4	15.7 ± 30.6	4.7 ± 7.1	0.003
Negative AFB smear^b ^(n = 143)	69.9 ± 76.2	53.3 ± 95.5	44.3 ± 42.8	20.1 ± 27.0	0.001
Laboratory diagnostic delay^2,b ^(n = 415)	2.6 ± 2.1	4.5 ± 3.1	3.5 ± 2.6	2.4 ± 2.4	< 0.001
Outcome; n (%)					
Successful treatment^a ^(n= 421)					0.001
Yes	73 (65.2)	99 (63.9)	68 (68.0)	50 (92.6)	
No	39 (34.8)	56(36.1)	32 (32.0)	4 (7.4)	
Fatality^a ^(n = 421)					0.002
Yes	31 (27.7)	50 (32.3)	28 (28.0)	3 (5.6)	
No	81 (72.3)	105 (67.7)	72 (72.0)	51 (94.4)	

AFB, acid-fast bacilli

^a^Using the chi-square test

^b^Using the Kruskal Wallis test

^1^Healthcare institution delay indicates days between the first related hospital visit and the initiation of anti-TB medication

^2^Laboratory diagnostic delay indicates workdays for reporting sputum smear examination results at the hospital

Most patients (74.1%) provided at least three sputum specimens for examination at the Chest Specialty Hospital; however, the proportion was only 48.2%, 36.8%, and 50.0% at medical centers, regional hospitals, and district hospitals/primary practitioners, respectively (*p *< 0.001) (Table 1). A positive sputum smear preceding a negative sputum culture result was more common at district hospitals/primary practitioners than at other institutions (*p *< 0.001).

Regarding efficiency in the diagnosis and treatment, the mean healthcare institution delay was 10.1 days at the Chest Specialty Hospital, which was shorter than those at medical centers, regional hospitals, and district hospitals/primary practitioners (31.0 days, 31.2 days, and 25.4 days, respectively) (*p *< 0.01, for all comparisons). Whereas the number of days between the chest X-ray examination and the hospital visit were similar, the number of days between the sputum examination and the hospital visit varied among different levels of healthcare institutions (*p *< 0.05 for both) and was shortest at the Chest Specialty Hospital. Furthermore, the Chest Specialty Hospital had the shortest mean laboratory diagnostic delay (2.4 days vs. 2.6 days, 4.5 days, and 3.5 days).

The Chest Specialty Hospital achieved a 92.6% successful treatment rate, which was higher than medical centers, regional hospitals, and district hospitals/primary practitioners (65.2%, 63.9%, and 68.0%, respectively, *p *< 0.01 for all comparisons) (Table 1). Moreover, the fatality rate at the Chest Specialty Hospital was the lowest among the institutions (5.6% vs. 27.7%, 32.3%, and 28.0%, *p *< 0.05 for all comparisons).

Full model and reduced model of multiple regression analyses revealed that patients of advanced age had less successful treatment rate, of which OR (95% CI) was 0.97 (0.96, 0.98). The patients who were treated by the pulmonologists and treated at Chest Specialty Hospital had more successful treatment rate, which OR were 1.74 (1.08, 2.97) and 4.58 (1.58, 13.23) respectively (Table 2). Meanwhile, we also discovered that amongst pulmonologists, those who provided services at the Chest Specialty Hospital had more successful treatment rate with OR 4.23 (1.45, 12.40) after adjusting age and gender (Table 3).

**Table 2:** Multiple logistic regression for factors affecting the successful treatment

		**Fullmodel**		**Reduced model^@^**	
			
**Variables**	**Reference group**	β	**OR (95% CI)**	β	**OR (95% CI)**
**Age**		-0.03	0.97 (0.96, 0.98)***	-0.03	0.97 (0.96, 0.98)***
**Gender**					
Female	Male	0.18	1.19 (0.72, 1.99)		
**Physician**					
Pulmonologist	Non-pulmonologist	0.54	1.72 (1.07, 2.77)*	0.55	1.74 (1.08, 2.79)*
**Institution**					
Chest Specialty Hospital	Other healthcare institutions	1.54	4.65 (1.61, 13.46)**	1.52	4.58 (1.58, 13.23)**
					
**Hosmer and Lemeshow**		X^2 ^= 7.25		X^2 ^= 8.57	

^@ ^reduced model using forward substitution; * *p *< 0.05, ** *p *< 0.01, *** *p *< 0.001

**Table 3:** Multiple logistic regression for factors affecting the successful treatment

		**Full model**		**Reduced model^a^**	
			
**Varia bles**	**Reference group**	β	**OR (95% CI)**	β	**OR (95% CI)**
**Age**		-0.04	0.96 (0.94, 0.98)***	-0.04	0.97 (0.94, 0.98)***
**Gender**					
Female	Male	0.29	1.33 (0.72, 2.47)		
**Pulmonologist at**					
Chest Specialty Hospital	Other healthcare institutions	1.44	4.23(1.45, 12.40)**	1.42	4.13 (1.41, 12.05)**
					
**Hosmer and Lemeshow**		X^2 ^= 9.06		X^2^=ll.96	

^a^reduced model using forward substitution; * *p *< 0.05, ** *p *< 0.01, *** *p *< 0.001

Furthermore, the patients of advanced age had more fatality rate, of which OR 1.06 (1.04, 1.09). The patients who were treated at Chest Speciatly Hospital had less fatality with OR 0.20 (0.06, 0.66) (Table 4).

**Table 4:** Multiple logistic regression for factors affecting the fatality rate

		**Full model**		**Reduced model^@^**	
			
**Variables**	**Reference group**	β	**OR (95% CI)**	β	**OR (95% CI)**
**Age**		0.06	1.06 (1.04, 1.08)***	0.06	1.06 (1.04, 1.08)***
**Gender**					
Female	Male	-0.12	0.89 (0.51, 1.55)		
**Physician**					
Pulmonologist	Non-pulmonologist	-0.36	0.70 (0.42, 1.162)		
**Institution**					
Chest Specialty Hospital	Other healthcare institutions	-1.54	0.21 (0.06, 0.73)*	-1.63	0.20 (0.06, 0.66)**
					
**Hosmer and Lemeshow**		X^2 ^= 7.25		X^2 ^= 4.40	

^@ ^reduced model using forward substitution; * *p *< 0.05, ** *p *< 0.01, *** p < 0.001

## Discussion

The persistence of infectious PTB as a major public health problem results from the presence of individuals with active infection in whom the disease remains undiagnosed or in whom a delay in diagnosis occurs [14-17]. The varied quality of healthcare provided by public and private sectors, and the failure of some healthcare providers to diagnose tuberculosis promptly and to ensure the delivery of adequate treatment are two major factors contributing to the poor control of tuberculosis [18]. Despite the fact that the NHI program has improved the accessibility of healthcare services to patients, tuberculosis cases continue to increase in Taiwan. This phenomenon prompted our investigation into the variations in care quality for infectious PTB patients at different levels within healthcare institutions.

In our study, male predominance and elderly susceptibility to infection by infectious PTB were observed, which are consistent with other published data [19-21]. Patients treated at the Chest Specialty Hospital were more likely to follow the international diagnostic guideline, which is the presentation of at least three sputum specimens for examination [9,10]. This might be due to more education and awareness of infectious PTB among patients cared for at the Chest Specialty Hospital. A higher proportion of positive sputum smears with subsequent negative sputum culture results was observed in district hospitals/primary practitioners. It is possible that district hospitals/primary practitioners are not as experienced as larger institutions or specialty institution. Thus, the laboratory performance at district hospitals/primary practitioners was not comparable to the quality at larger institutions. Poor laboratory diagnoses may affect the prescribing of anti-TB medications by physicians, which in turn may adversely affect the patient's safety [22]. Sputum smear microscopy is a simple, inexpensive, and appropriate technique for diagnosing tuberculosis, and laboratories in healthcare institutions should have qualified technicians performing this technique [8,9]. Failing this, sputum examinations should be referred to the central laboratories responsible for testing sputum specimens in each district [23].

The present study has shown that laboratory diagnostic delay and healthcare institution delay, regardless of acid-fast smear, were shorter at the Chest Specialty Hospital, which is most likely due to its greater vigilance for infectious PTB detection. However, even this prompt care cannot be initiated until a patient has sought or been referred for treatment. In this regard, patients themselves contributed to seeking treatment, and such delays were independent of the healthcare institution. Further extensive public health education programs may be necessary to strengthen the public awareness of infectious PTB and thereby decrease patient delay in seeking care.

Efficient diagnosis and prompt treatment are the key components to the effective control of infectious PTB [14,24,25]; and, according to practice guidelines for the treatment of tuberculosis [10], the rate of successful treatment is a good performance indicator of diagnosis and treatment. The high successful treatment rate observed at the Chest Specialty Hospital (up to 92.6%), moreover, after adjusting age and gender, the patients treated at Chest Specialty Hospital still had more successful treatment rate and less fatality rate. That might be due to the physicians' expertise with the disease and more efficient diagnostic and treatment process. Patients treated by pulmonologists appeared to have a higher successful treatment rate than patients treated by non-pulmonologist physicians. Perhaps a pivotal factor lies within the unfamiliarity non-pulmonologist physicians have with the treatment of TB patients. Furthermore, the fact that patients treated by pulmonologists at the Chest Specialty Hospital had a higher successful treatment rate than those who were treated by pulmonologists at other institutions might indicate that both training of care providers and the institution providing the care are important factors affecting the quality of care. Whereas clinical status and severity of the disease might affect successful treatment rate and fatality rates, no variables are available in the database we obtained to address this issue. Therefore, we were unable to evaluate the effects of these factors to the results, which could be a major limitation of the current study. Issues regarding patient delays have previously been studied [26-28]. The aim of this study was to identify variations in the quality of diagnosis and treatment of infectious PTB patients at different levels within healthcare institutions, and we found that both training of care providers and the institution providing the care are important factors affecting the quality of care. Our study showed that non-adherence to a consistent approach to the diagnosis and treatment for PTB at different levels within healthcare institutions might result in various successful treatment rates and that although pulmonologists provided better care than other physicians, the successful treatment rates achieved seemed different between pulmonologists at the Chest Specialty Hospital and pulmonologists at other institutions. In order to promote the quality of infectious PTB patient care in Taiwan to the same level as that at the Chest Specialty Hospital, extensive education programs and implementation of practice guidelines for tuberculosis for medical staff at the other institutions are strongly recommended. Furthermore, it is recommended that the healthcare institutions should also monitor their own diagnostic quality and the efficiency and effectiveness of treatment to patients.

## Conclusion

Differences in quality of care exist among different types of healthcare institutions and among individual physicians. The implementation of practice guidelines should contribute to an improvement in the quality of care in the treatment and diagnosis of PTB.

## List of abbreviations

PTB pulmonary tuberculosis

WHO World Health Organization

CDC Center for Disease Control

AFB acid-fast bacilli

## Competing interests

The author(s) declare that they have no competing interests.

## Authors' contributions

WSC and REC contributed to conception and design, acquisition of data, analysis and interpretation of data, and were involved in drafting the manuscript. HRG devoted himself to revising it and providing critical and important intellectual content. All authors read and approved the final manuscript.

## Pre-publication history

The pre-publication history for this paper can be accessed here:


